# Receptor and post-receptor abnormalities contribute to insulin resistance in myotonic dystrophy type 1 and type 2 skeletal muscle

**DOI:** 10.1371/journal.pone.0184987

**Published:** 2017-09-15

**Authors:** Laura Valentina Renna, Francesca Bosè, Sara Iachettini, Barbara Fossati, Lorenzo Saraceno, Valentina Milani, Roberto Colombo, Giovanni Meola, Rosanna Cardani

**Affiliations:** 1 Laboratory of Muscle Histopathology and Molecular Biology, IRCCS-Policlinico San Donato, San Donato Milanese, Milan, Italy; 2 Department of Neurology, IRCCS-Policlinico San Donato, San Donato Milanese, Milan, Italy; 3 Department of Biomedical Sciences for Health, University of Milan, Milan, Italy; 4 Scientific Directorate, IRCCS Policlinico San Donato, San Donato Milanese, Milan, Italy; 5 Department of Biosciences, University of Milan, Milan, Italy; Institut de Myologie, FRANCE

## Abstract

Myotonic dystrophy type 1 (DM1) and type 2 (DM2) are autosomal dominant multisystemic disorders caused by expansion of microsatellite repeats. In both forms, the mutant transcripts accumulate in nuclear foci altering the function of alternative splicing regulators which are necessary for the physiological mRNA processing. Missplicing of insulin receptor (IR) gene (*INSR*) has been associated with insulin resistance, however, it cannot be excluded that post-receptor signalling abnormalities could also contribute to this feature in DM. We have analysed the insulin pathway in skeletal muscle biopsies and in myotube cultures from DM patients to assess whether downstream metabolism might be dysregulated and to better characterize the mechanism inducing insulin resistance. DM skeletal muscle exhibits alterations of basal phosphorylation levels of Akt/PKB, p70S6K, GSK3β and ERK1/2, suggesting that these changes might be accompanied by a lack of further insulin stimulation. Alterations of insulin pathway have been confirmed on control and DM myotubes expressing fetal *INSR* isoform (*INSR-A*). The results indicate that insulin action appears to be lower in DM than in control myotubes in terms of protein activation and glucose uptake. Our data indicate that post-receptor signalling abnormalities might contribute to DM insulin resistance regardless the alteration of *INSR* splicing.

## Introduction

Myotonic dystrophy type 1 (DM1) and type 2 (DM2) are dominantly inherited neuromuscular disorders characterized by a multisystemic involvement including muscle hyperexcitability (myotonia), progressive muscle wasting, cardiac conduction defects, cataracts, neuropsychiatric disturbances and metabolic dysfunctions such as insulin resistance, hyperinsulinemia, hypertriglyceridemia [1–2]. DM1 is caused by an expanded CTG repeat in the 3′ untranslated region (UTR) of the *Dystrophia Myotonica Protein Kinase* gene (*DMPK*) [3–5], while DM2 is caused by expanded CCTG repeats in intron 1 of the *CCHC-type zinc finger*, *Nucleic acid Binding Protein* gene (*CNBP*/*ZNF9*) [6]. Nuclear accumulation of CUG/CCUG-containing RNA from the expanded allele is thought to contribute significantly to pathogenesis since these nuclear aggregates deregulate several splicing factors promoting the alteration of alternative splicing of various genes that have been linked to symptoms of DM [7–12].

Insulin resistance is the principal metabolic abnormality associated with DM pathology. DM patients present marked hyperinsulinemia after oral glucose tolerance testing even if they show normal basal insulin levels and normal glucose tolerance [13–15]. Impaired glucose utilization has been reported in several tissues from DM subjects [16–18]. It has been reported that glucose uptake in the forearm muscle remains three times lower in DM than in normal subjects after insulin intrabrachial arterial infusion from low to supraphysiological doses [19].

Insulin resistance is an important factor for the development of Type 2 Diabetes Mellitus (T2DM), and a risk factor for atherosclerosis, hypertension, cardiovascular diseases, obesity and loss of muscle mass [20–21]. Patients with DM are characterized by a fourfold higher risk of developing T2DM [22] and cardiac manifestations are among the most common systemic features of myotonic dystrophies accounting for approximately the 30% of the deaths in this population [23–24]. Moreover, DM patients are characterized by muscle weakness and atrophy and by skeletal muscle histopathological features that include fibre atrophy-hypertrophy, increased number of central nuclei, and presence of fibres with nuclear clumps [25–27]. Thus, insulin resistant state and metabolic changes in DM patients might contribute to worsen some multisystemic features of the disease in particular in heart, skeletal muscle and brain. In DM patients insulin resistance has been associated with aberrant splicing of the insulin receptor gene *(INSR)* due to a toxic effect of the CUG/CCUG-expanded repeats [10–11] that leads to a higher expression of the fetal isoform A (*INSR-A*, lacking exon 11) than the adult insulin-sensitive isoform B (*INSR-B*). It has been reported that DM1 differentiated skeletal muscle cells show a lower insulin responsiveness with regard to glucose uptake suggesting a strong correlation between an *INSR* abnormal splicing switch and a resistance to the metabolic effects of insulin [10, 28]. However, whether the entire endocrine pathology of DM is caused by alterations in *INSR* RNA processing remains to be explored and post-receptor defects in insulin signalling have been suggested [29–31]. Recently, Jones and collaborators [32] reported high levels of glycogen synthase kinase-3 beta (GSK3β) in skeletal muscle from DM1 patients that cause a reduction of cyclin D3 expression. GSK3β is a protein kinase involved in glucose homeostasis whose activity is inhibited by phosphorylation upon insulin stimulation and it is increased in muscle of T2DM patients [32–37]. Moreover, the authors reported that the increase in the activity of GSK3β in DM patients was linked to a reduction in the levels of p68, a RNA helicase involved in dsRNA unwinding thus allowing ribonucleases to access RNA-binding sites and facilitate RNA degradation. The correction of p68 expression led to degradation of the mutant RNAs in both DM1 and DM2 cells and intramuscular injection of p68 in HSA^LR^ mice improved muscle histopathology [38]. Gao and Cooper [39] reported a substantial increase of pyruvate kinase M2 (PKM2), a critical glycolytic enzyme, in DM1 skeletal and cardiac muscle tissue. Moreover, PKM2 was selectively up-regulated in type 1 muscle fibers, which are susceptible to atrophy in DM1 patients supporting the hypothesis that altered glucose metabolism may contribute to DM1 skeletal muscle pathomolecular mechanism. More recently, it has been reported that in HSA^LR^ mice AMPK/mTORC1 signalling, which are central metabolic pathways in muscle cells, were impaired in skeletal muscle. Since in HSA^LR^ mice no changes in *INSR* alternative splicing were reported, AMPK/mTORC1 deregulation appears to be independent of insulin receptor deficiency [40].

The two best characterized insulin signalling pathways are the Ras-ERK (extracellular-signal-regulated kinase) pathway and the IRS (insulin receptor substrate)-Akt/PKB (protein kinase B) pathway. The actions of these two pathways are different, with the Ras-ERK pathway primarily involved in growth and proliferation [41], and the IRS-PKB pathway involved in many actions of insulin such as regulation of glucose transport [42–43], gluconeogenesis [44], glycogen synthesis [45] and also in the control of growth and apoptosis [46]. The aim of this study was to determine whether changes in expression of key proteins in these two insulin-signalling pathways and in glucose transport account for impaired insulin action in skeletal muscle from DM1 and DM2 patients. Indeed, as skeletal muscle is responsible for the majority of the body post-prandial glucose disposal, insulin resistance in this tissue results in substantial whole body metabolic disturbances. Since it has been reported that DM1 shows greater biomolecular alterations in distal than in proximal muscles and that the reverse occurs for DM2 [11, 47], both *tibialis anterior* (TA) and *biceps brachii* (BB) were analysed in this study.

The study of the molecular mechanisms that induce insulin resistance in both DM1 and DM2 patients could lead to identify novel biomarkers that will be target for therapeutic interventions to treat metabolic dysfunctions and thus improve the quality of life of DM patients.

## Materials and methods

The study protocol was reviewed and approved by the ethical committee Ospedale San Raffaele (Milan, Italy) and was conducted according to the principles expressed in the Declaration of Helsinki, the institutional regulation and Italian laws and guidelines. Written informed consents were obtained from the patients for all blood samples and muscle biopsies used in this study.

### Patients and muscle biopsies

*Biceps brachii* (BB) and *tibialis anterior* (TA) muscle biopsies were taken under sterile conditions from a total of 8 DM1 (6 BB, 4 TA) and 5 DM2 (5 BB) patients enrolled in The Italian Registry for Myotonic Dystrophy Type 1 and Type 2. Among DM1, 2 patients had two successive biopsies at different years of age: first BB biopsy and, 7–8 years later, also had a research biopsy of TA. In DM2 patients, BB biopsies were performed for diagnostic purposes while in DM1 both BB and TA muscle biopsies were obtained for research intents. Eight age-matched subjects with no sign of neuromuscular disease (5 BB, 3 TA) were used as controls (CTR). DM2 and CTR patients were randomly selected among subjects who performed a diagnostic muscle biopsy, while 8 DM1 patients were recruited from those registered in The Italian Registry for Myotonic Dystrophy Type 1 and Type 2. All patients underwent overnight fasting before blood and muscle samples collection. Muscle tissue was fresh-frozen in isopentane cooled in liquid nitrogen. Histopathological analysis was performed on serial sections (8 μm) processed for routine histological or histochemical stainings. The diagnosis of DM was based upon the clinical diagnostic criteria set by the International Consortium for Myotonic Dystrophy [48]. DM2 diagnosis was performed by fluorescence in situ hybridization on muscle frozen sections using a (CAGG)_5_ probe as previously reported by Cardani et al. [49] to verify the presence of nuclear accumulation of mutant RNA. DM1 genotyping was performed on genomic DNA extracted from peripheral blood leukocytes as previously described [50].

### Immunohistochemistry

Immunohistochemical staining was performed on serial sections (6 μm) air-dried and rehydrated in phosphate buffer solution pH 7.4 (PBS). Non-specific binding sites were blocked with normal goat serum (NGS; Dako, Glostrup, Denmark) at a dilution 1:20 in PBS containing 2% bovine serum albumin (BSA; Sigma-Aldrich, St. Louis, MO, USA) for 20 min at room temperature (RT). Sections were then incubated for 1 h with mouse monoclonal primary antibodies against two different myosin heavy chain (MHC): MHCfast (1:400 in PBS+2%BSA, Sigma-Aldrich) and MHCslow (1:400 in PBS+2%BSA, Sigma-Aldrich). Sections were washed in PBS (3x5 min) and then incubated for 1 h with goat anti-mouse biotinylated secondary antibody diluted 1:300 in PBS+2%BSA. After washing in PBS (3x5 min), sections were incubated for 30 min with Vectastain ABC complex (Vector Laboratories, Burlingame, CA, USA) and then with 3,3’-Diaminobenzidine (DAB) and hydrogen peroxide for 20 min. Finally, nuclei were counterstained with Mayer’s hematoxylin. Quantitative evaluation of fiber diameter was made as described previously by Vihola et al. [27] using Image J (Scion Co.) on images taken with a light microscope (original magnification 200x).

### Primary skeletal muscle cell cultures

The human satellite cells-derived myoblasts were isolated from DM and CTR muscle biopsies as previously reported by Cardani et al. [51]. Myogenic purity and differentiative capability were evaluated [51]. Myoblasts obtained from BB biopsy of two T2DM patients were used as internal control. Myoblasts were grown in Dulbecco Modified Eagle Medium (D-MEM, Sigma-Aldrich, St. Louis, MO) high glucose (4,5 g/L glucose) supplemented with 15% FBS (Euroclone, Milan, Italy), 0.5 mg/mL albumin from bovine serum (BSA, Sigma-Aldrich, St. Louis, MO), 0.5 mg/mL fetuin (Sigma-Aldrich, St. Louis, MO), 0.39 μg/mL dexamethasone (Sigma-Aldrich, St. Louis, MO), 10 ng/mL epidermal growth factor (Sigma-Aldrich, St. Louis, MO), 2 mM L-glutamin (Euroclone, Milan, Italy), 100 U/mL penicillin and 100 μg/mL streptomycin (Euroclone, Milan, Italy) (proliferative medium) at 37°C in a 5% CO_2_-95% air atmosphere. All cell populations used in this study had a myogenic purity higher than 80%. When myoblasts reached a confluence of 80%, they were induced to differentiate in myotubes replacing the proliferative medium with D-MEM high glucose supplemented with 7% FBS, 2 mM L-glutamin, 100 U/mL penicillin and 100 μg/mL streptomycin (differentiative medium). After 5 days of differentiation (T5), cells were serum starved with D-MEM low glucose (1 g/L glucose) supplemented with 2 mM L-glutamin, 100 U/mL penicillin and 100 μg/mL streptomycin for 16 hours and then stimulated with 10 nM insulin (Insulin aspart, NovoRapid). The differentiative capability was similar among all samples analysed.

### Alternative splicing of insulin receptor

The *INSR* alternative splicing was investigated both in muscle biopsies and in myotubes. Muscle biopsies obtained from 5 DM1 (4 BB and 3 TA) and 3 DM2 (3 BB) patients and from 5 CTR (3 BB and 2 TA) patients were mechanically lysed in 1 ml of TRIzol reagent (Gibco BRL, Gaithersburg, MD) using Tissue Lyser (Qiagen). To analyse the *in vitro INSR* alternative splicing, myotubes from 3 DM1, 3 DM2, 1 T2DM and 3 CTR subjects were differentiated for 5 days and then harvested and practiced for the extraction of total RNA using TRIzol reagent (Gibco BRL, Gaithersburg, MD).

1 μg of total RNA was reverse transcribed to cDNA using SuperScript III First-Strand Synthesis System (Invitrogen, Thermo Scientific, Rockford, USA) following manufacturer’s instruction. The *INSR* gene was amplified by classical RT-PCR using gene specific primers: forward primer 5’-CCAAAGACAGACTCTCAGAT-3’ and reverse primer 5’-AACATCGCCAAGGGACCTGC-3’. Each PCR reaction was performed in triplicate using Platinum Taq (Thermo Scientific, Rockford, USA) according to manufacturer’s protocol. Total RT-PCR products were electrophoretically resolved on 2,5% agarose gel. Qualitative analysis of the alternative splicing of *INSR* was performed using EtBr-stained gel (Sigma-Aldrich, St. Louis, MO) scanned on a ChemiDoc Universal Hood (BioRad). Quantitative analysis was performed quantifying the intensity of each band with ImageJ software densitometry and calculating the proportions of normally spliced (*INSR-B*) isoform respect to the total amount of the isoforms.

### Protein extraction and western blot analysis

*In vivo* basal expression of several proteins of insulin pathway has been analysed in 6 DM1, 5 DM2 and 8 CTR muscle biopsies. Whole cell protein extracts were obtained from twenty consecutive muscle cryostat sections 10 μm thick homogenized in 60 μl of 50 mM TrisHCl with 5% SDS (pH 7.5). After incubating on ice for 15 min, samples were centrifuged at 5700 g for 15 min at 4°C and supernatant was collected and stored at -80°C.

To investigate the response of skeletal muscle cells to insulin stimulation, T5 myotubes (5 DM1, 5 DM2 and 6 CTR) were serum starved for 16 hours and then stimulated with 10 nM of insulin for 0, 5, 15, and 30 minutes. Cells were harvested, washed in PBS and then lysed on ice for 15 min in a buffer containing 50mM Tris HCl pH 7.5, 150 mM NaCl, 1 mM EDTA, 1% NP40 and supplemented with protease and phosphatase inhibitors. Samples were then centrifuged at 5700 g for 15 minutes at 4°C and supernatant was collected and stored at -80°C.

Protein concentration was determined with Pierce BCA Assay Kit (Thermo Scientific, Rockford, USA). 40 μg of total proteins were separated by SDS-PAGE and transferred to nitrocellulose membranes. After blocking non-specific binding with TrisHCl buffer pH 7.5 (TBS) containing 5% BSA, the membranes were incubated with primary antibodies. After washing with TBS+0.3% Tween20, membranes were incubated with HRP-coniugated anti-mouse or anti-rabbit secondary antibodies (Jackson ImmunoResearch Laboratories, INC) diluted 1:5000 or 1:10000 in TBS+5%BSA+0,2%Tween20 respectively. Super signal West Pico Chemiluminescent Substrate (Thermo Scientific, Meridian Rd., Rockford, USA) was used for immunodetection. For western blot application, the following primary antibodies were used: rabbit monoclonal anti-Insulin Receptor β (4B8), which recognises both IR-A and IR-B isoforms, (Cell Signaling Technology, 1:500); rabbit polyclonal anti-IRS1 phospho Tyr896 (GeneTex; 1:400); rabbit polyclonal anti-IRS1 phospho Tyr612 (Abcam, 1:300); rabbit polyclonal anti-IRS1 (GeneTex; 1:500); mouse monoclonal anti-Akt phospho Thr308 (clone L32A4, Cell Signaling Technology; 1:500); rabbit polyclonal anti-Akt (Cell Signaling Technology; 1:2000); rabbit polyclonal anti-p70S6 kinase phospho Thr421/Ser424 (Cell Signaling Technology; 1:1000); rabbit polyclonal anti-p70S6 kinase (Cell Signaling Technology; 1:500); rabbit polyclonal anti-GSK3b phospho Tyr216 (Santa Cruz Biotechnology, INC; 1:400); mouse monoclonal anti-GSK3β (clone 1V001, Santa Cruz Biotechnology, INC; 1:500); rabbit polyclonal anti-p44/42 MAPK (Erk1/2) phospho Thr202/Tyr204 (Cell Signaling Technology; 1:1000); mouse monoclonal anti-p44/42 MAPK (Erk1/2) (clone 3A7, Cell Signaling Technology; 1:2000). All the phosphorylated isoforms analysed in this study correspond to an active form of the protein. Rabbit polyclonal anti-GAPDH (Sigma—Aldrich, St. Louis, MO; 1:20000) was used as an internal loading control. Each experiment was performed in triplicate and quantitative analysis was performed quantifying the intensity of each band with ImageJ software densitometry. Since samples were run on different gels, the same CTR samples were loaded on all gels performed as internal control to allow normalization after antibody probing.

### Glucose uptake analysis

The capability of myotubes (T5) to uptake glucose was performed using the Glucose uptake colorimetric assay kit (Biovision) according to manufacturer’s instruction.

### Statistical analysis

Statistical analysis was performed using SAS software, version 9.4 (SAS Institute, Inc., Cary, NC). For comparison between two groups Student’s *t*-test was used. For evaluation of *in vitro* insulin signalling activation in CTR and DM muscle cells, one-way ANOVA (repeated measures) was used to evaluate the effect of time within groups. Post-hoc test was performed. The differences were considered statistically significant at p<0.05.

## Results

### Patients

This study was performed on a total of 8 DM1 and 5 DM2 patients compared to 8 age-matched subjects with no sign of neuromuscular disease used as controls (CTR). The DM1 cohort was represented by classical adult patients (mean ± standard deviation of CTG repeat expansion 370.6±111.1); the DM2 cohort was represented by patients with classical PROMM (Proximal Myotonic Myopathy) phenotype. Data on CTR and DM patients are reported in Table 1 and S1 Table. Among DM2, one patient (DM2-1) was affected by T2DM and treated with metformin. BMI (Body Mass Index) was similar between CTR and DM1 patients, while it was slightly higher in DM2 patients compared to CTR. As control for *in vitro* study of activation of insulin pathway, 2 patients affected by T2DM (T2DM-1, male 54 years old; T2DM-2, male 50 years old) were used. T2DM-1 patient was treated with insulin. To allow the comparison between data obtained on skeletal muscle biopsies, all samples were taken in the morning after overnight fasting.

**Table 1:** Clinical data on CTR and DM patients used in this study.

**Patient**	**Sex**	**Muscle**	**Age at onset**	**Age at biopsy (I/II)**^a^	**CTG repeat size**	**MRC**^b^ **(I/II)**	**MIRS**^c^ **(I/II)**
**CTR-1**	F	BB	-	42	-	n.d	-
**CTR-2**	M	BB	-	47	-	n.d	-
**CTR-3**	M	BB	-	35	-	n.d	-
**CTR-4**	M	TA	-	27	-	n.d	-
**CTR-5**	M	TA	-	27	-	n.d	-
**CTR-6**	F	TA	-	30	-	n.d	-
**CTR-7**	F	BB	-	22	-	n.d	-
**CTR-8**	F	BB	-	49	-	n.d.	-
**DM1-1**	F	BB/TA	35	45/52	460	127.3/102.7	4/5
**DM1-2**	F	BB	24	28	370	138.3	3
**DM1-3**	M	BB	25	49	255	137,3	3
**DM1-4**	F	BB/TA	20	22/30	560	148.7/146.7	2/2
**DM1-5**	F	TA	22	33	320	144.6	3
**DM1-6**	F	TA	31	35	350	145.1	3
**DM1-7**	F	BB	20	48	430	136.4	4
**DM1-8**	F	BB	22	44	220	113.21	3
**DM2-1**	F	BB	54	59	-	120.9	-
**DM2-2**	F	BB	35	53	-	138.6	-
**DM2-3**	M	BB	31	34	-	148.6	-
**DM2-4**	F	BB	58	61	-	143	-
**DM2-5**	F	BB	31	36	-	148.7	-
**Patient**	**Serum Total Cholesterol (mg/dl: <200) (I/II)**		**Fasting Serum Glucose (mg/dl:70–110) (I/II)**		**Fasting Serum Insulin (μU/ml: 2.6–24.9) (I/II)**	**HOMA**^d^ **(<2.5) (I/II)**	**BMI**
**CTR-1**	156		79		n.d.	n.d.	18.4
**CTR-2**	145		85		n.d.	n.d.	26.0
**CTR-3**	194		90		n.d.	n.d.	24.9
**CTR-4**	132		100		n.d.	n.d.	24.5
**CTR-5**	140		96		n.d.	n.d.	25.0
**CTR-6**	135		94		n.d.	n.d.	20.7
**CTR-7**	132		89		n.d.	n.d.	20.2
**CTR-8**	134		82		n.d.	n.d.	22.1
**DM1-1**	245/207		89/85		12.9/14.0	2.85/2.9	24.97/27.06
**DM1-2**	143		79		4.8	0.93	22.2
**DM1-3**	133		79		4.3	0.84	24.3
**DM1-4**	166/149		76/83		n.d/10.44.	n.d/2.13	20.2/20.3
**DM1-5**	140		95		11.79	2.7	23.4
**DM1-6**	238		81		8.59	1.71	22.8
**DM1-7**	154		73		12.63	2.2	28.7
**DM1-8**	189		90		10.2	2.2	22.1
**DM2-1**	226		110		28.3	7.68	n.d.
**DM2-2**	239		90		27.7	6.15	28.9
**DM2-3**	222		80		3.0	0.59	25.0
**DM2-4**	229		81		9.71	1.8	24.4
**DM2-5**	163		88		5.87	1.1	20.7

CTR, control; DM1, myotonic dystrophy type 1; DM2, myotonic dystrophy type 2; F, female; M, male.

^a^Clinical data reported are relative to the time at the first biopsy (I) and at the second biopsy (II)

^b^Medical Research Council, scale for muscle strength; scale (0–5 grade) on 15 muscles at both sides in the upper and lower limbs for a total of 150 maximum score.

^c^Muscle Impairment Rating Scale, stage of the disease for myotonic dystrophy type 1 (DM1) patients [52].

^d^HOMA (HOmeostasis Model Assessment) was calculated using the formula: HOMA = [glucose (mg/dl) x insulin (μU/mL)/405], using fasting values.

### Muscle histopathology

**Fig 1:**
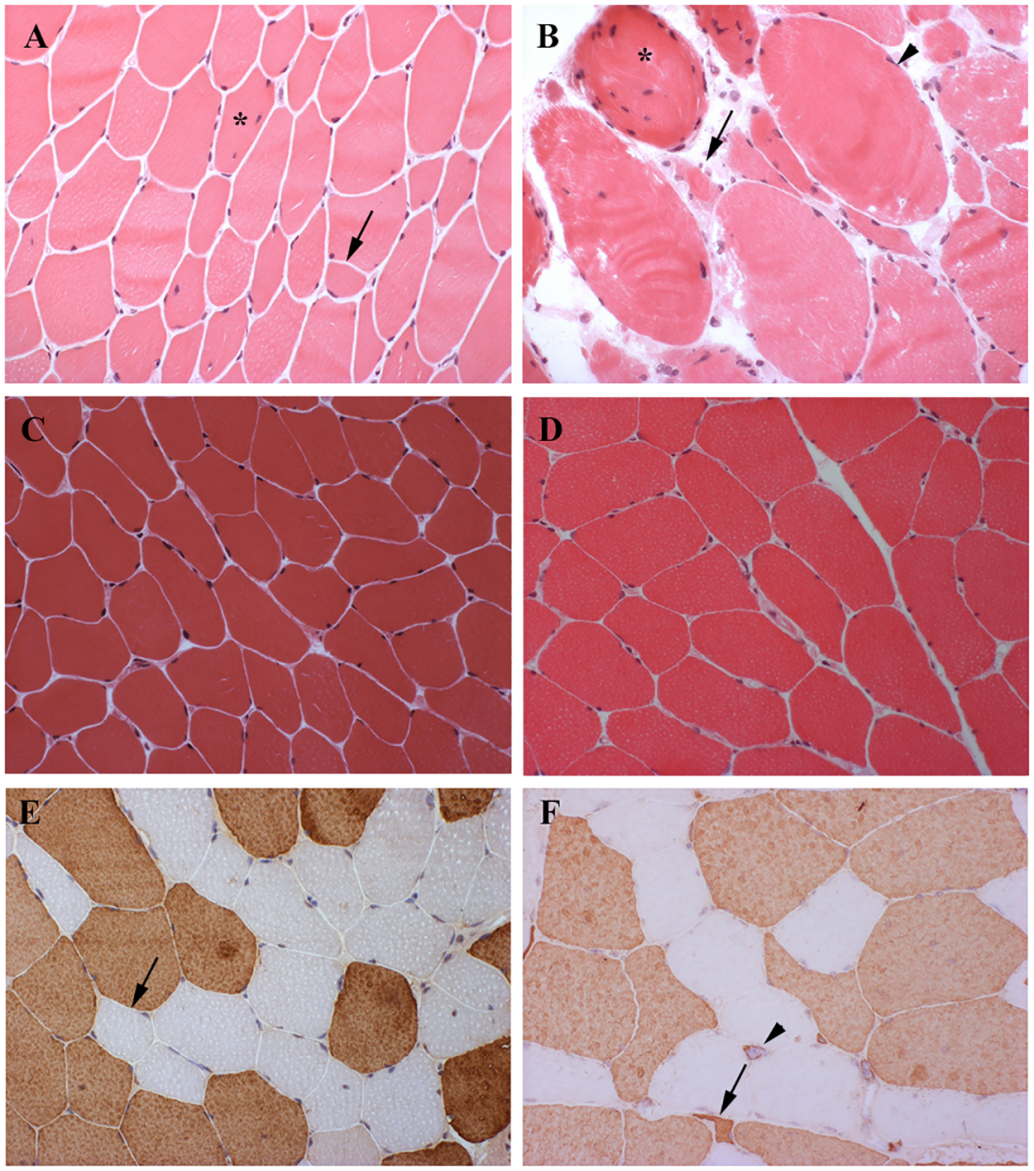
Histopathological analysis of muscle biopsies. **(A-D)** H&E staining of muscle sections obtained from two DM1 patients who performed two successive biopsies at different years of age. In the first *biceps brachii* biopsy (A) of DM1-1 patient a variation in fiber size, central nuclei (asterisk) and atrophic fibers (black arrow) were present, while the second *tibialis anterior* biopsy (B) was severely affected with end-stage changes including loss of muscle fibers, fibrosis, marked increase in the number of fibers with internal nuclei (asterisk) and fiber hypertrophy (arrow head) and atrophy (black arrow). In DM1-4 patient, the first *biceps brachii* biopsy (C) and the second *tibialis anterior* biopsy (D) showed minimal myopathic changes. **(E, F)** Fast myosin immunostaining of *biceps brachii* muscle sections obtained from DM1-2 (E) and from DM2-2 patients (F). Atrophic fibers were prevalently of type 1 in DM1 section (E, black arrow) and of type 2 in the DM2 section (F, black arrow). Nuclear clumps fast myosin positive were also present in DM2 section (F; arrow head). Original magnification 200x.

Routine stainings performed on BB and TA transverse sections revealed the presence of the histological alterations commonly observable in DM skeletal muscle such as nuclear clump fibers, nuclear centralization, fiber size variability and fibrosis (Fig 1A–1F). Moreover, the immunohistochemical staining of MHC slow and fast myosin allowed to better evaluate type 1 and type 2 fiber atrophy and hypertrophy and to verify the dystrophic changes (Fig 1E and 1F). As expected, a predominant involvement of type 1 fibers in DM1 and a prevalent involvement of type 2 fibers in DM2 was observed. The results of the histopathological analysis of the routine stained sections and of immunostained sections are reported in Table 2. Two DM1 patients had two successive muscle biopsies for research purposes (Table 1). DM1-1 patient had her first BB biopsy at the age of 45 and the muscle tissue already showed typical DM1 features such as fiber size variability, central nuclei and type 1 fiber atrophy (Table 2; Fig 1A). The second TA biopsy was performed 7 years later and it showed extensive end stage changes with dystrophic features (fibrosis) and marked increase in the number of fibers with internal nuclei, fiber hypertrophy and type 1 fiber atrophy (Table 2; Fig 1B). In DM1-4 patient, the BB biopsy was performed at the age of 22 and the second TA biopsy at the age of 30. In both biopsies, minimal myopathic changes were present (Table 2; Fig 1C and 1D).

**Table 2:** Histopathological features of skeletal muscle in DM patients.

Patient	Muscle	Central nuclei (I/II)^a^	Nuclear clumps (I/II)	AF fast (I/II)	AF slow (I/II)	HF fast (I/II)	HF slow (I/II)	Fibrosis (I/II)
**DM1-1**	BB/TA	<10%/>20%	absent/present	-/+	+/++	+/++	-/++	±/++
**DM1-2**	BB	<5%	absent	-	-	++	+	-
**DM1-3**	BB	10%-20%	absent	+	++	+	+	-
**DM1-4**	BB/TA	<5%/<10%	absent/absent	-/-	+/±	±/±	±/±	-/-
**DM1-5**	TA	10%-20%	absent	-	++	+	+	-
**DM1-6**	TA	10%-20%	absent	+	+	+	+	-
**DM1-7**	BB	10%-20%	present	±	+	+	+	-
**DM1-8**	BB	>20%	absent	+	++	+	+	±
**DM2-1**	BB	<10%	numerous	++	-	-	-	-
**DM2-2**	BB	>20%	numerous	++	-	+	+	-
**DM2-3**	BB	<10%	absent	-	-	+	+	-
**DM2-4**	BB	>20%	numerous	++	-	+	-	-
**DM2-5**	BB	>20%	present	+	-	+	-	-

AF fast, fast fiber atrophy; AF slow, slow fiber atrophy; HF fast, fast fiber hypertrophy; HF slow, slow fiber hypertrophy; -, absent; ±, modest; +, present; ++, prominent (grade based on fiber diameter analysed on immunostained sections).

^a^Data are referred to the first biopsy (I) and to the second biopsy (II).

### Insulin receptor alternative splicing and insulin signalling in muscle biopsies from DM patients

**Fig 2:**
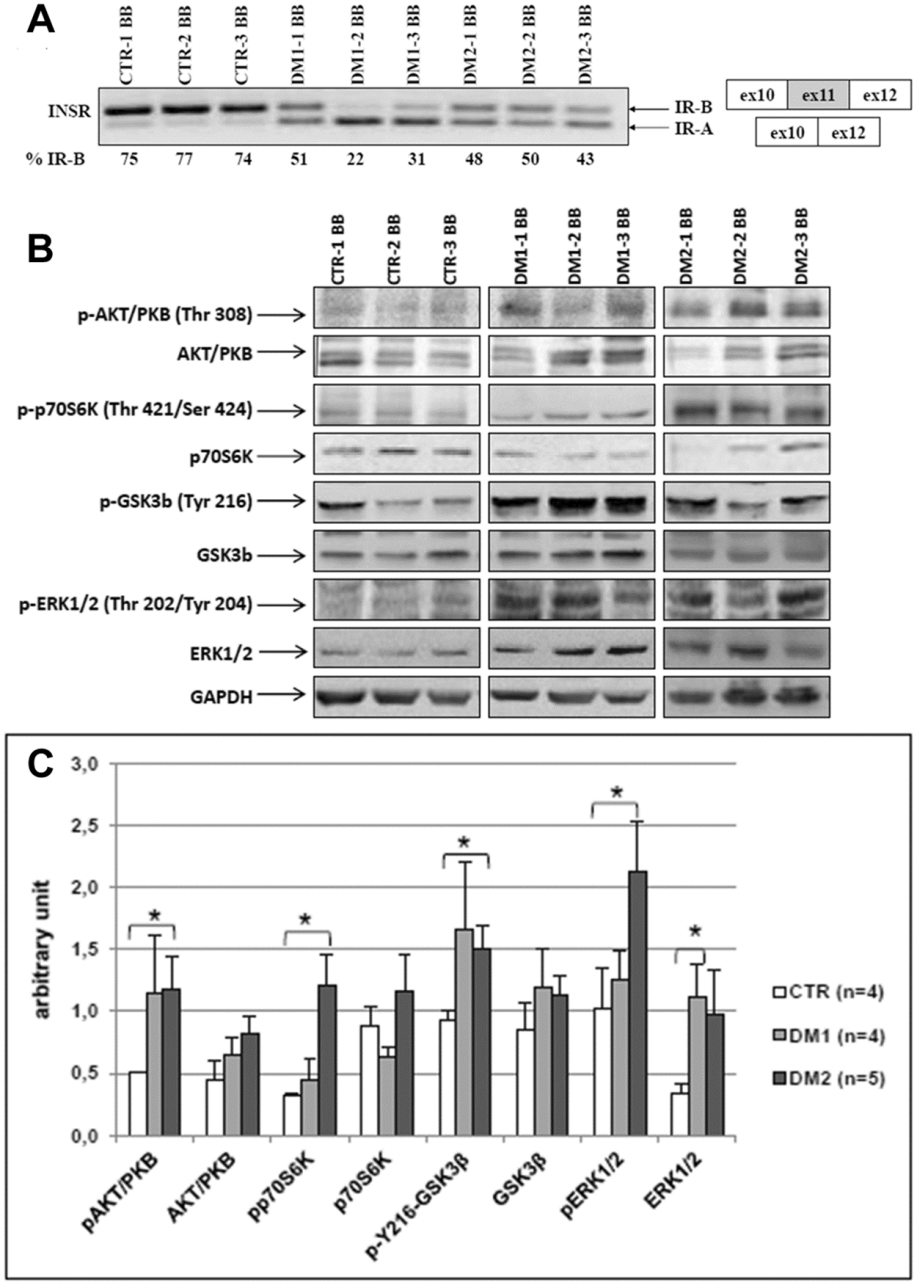
Insulin receptor alternative splicing and insulin signalling in *biceps brachii* muscles. **(A)** *INSR* splicing products obtained by RT-PCR amplification of RNA isolated from *biceps brachii* biopsies obtained from CTR, DM1 and DM2 patients. Bands were quantified and proportions of isoform *INSR-B* (+exon 11) were calculated. (**B)** Representative western blot analysis of the expression of proteins involved in the insulin pathway in CTR, DM1 and DM2 patients. Due to the high number of samples analysed, the figure panel is composed by images of bands originating from different gels. Details on how western blot experiments were performed are reported in Materials and Methods. (**C)** Quantification of proteins expression normalized to GAPDH. Histograms represent mean values and bars represent standard error of the mean (SEM). The number of samples analysed in each group (n) is reported in histogram legend. Differences between groups have been evaluated by Student *t*-test. *p<0.05.

**Fig 3:**
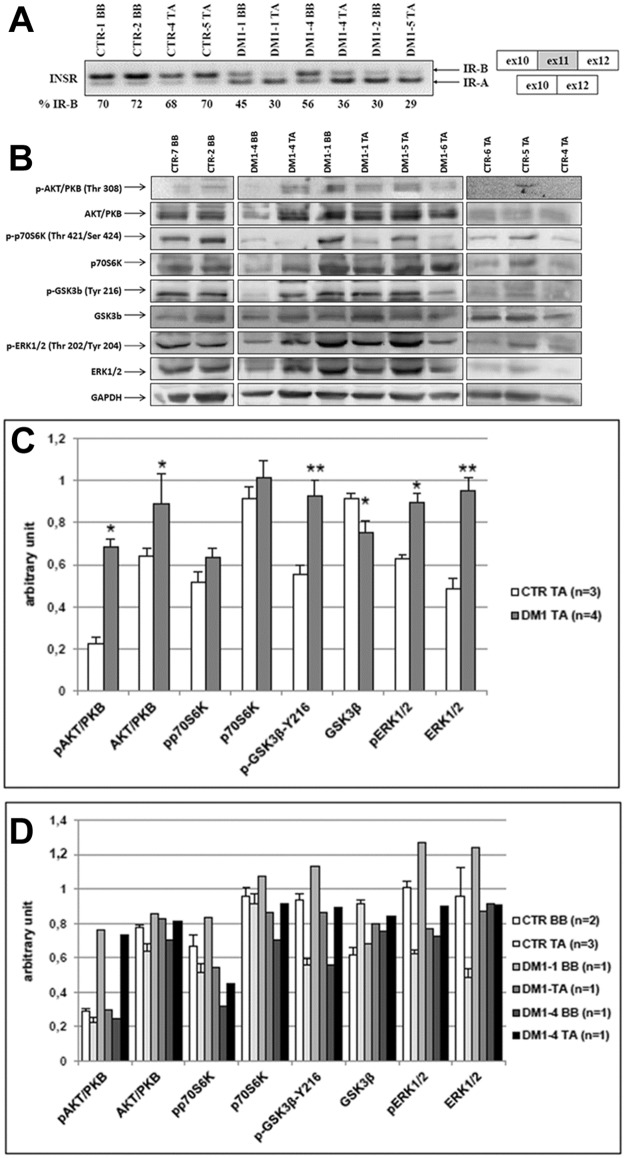
Insulin receptor alternative splicing and insulin signalling in *biceps brachii* and *tibialis anterior* muscles of DM1 patients. **(A)** *INSR* splicing products obtained by RT-PCR amplification of RNA isolated from *biceps brachii* and *tibialis anterior* biopsies obtained from CTR and DM1 patients. Bands were quantified and proportions of isoform *INSR-B* (+exon 11) were calculated. (**B)** Representative western blot analysis of the expression of proteins involved in the insulin pathway in *biceps brachii* and *tibialis anterior* biopsies obtained from CTR and DM1 patients. Due to the high number of samples analysed, the figure panel is composed by images of bands originating from different gels. Details on how western blot experiments were performed are reported in Materials and Methods. (**C)** Quantification of proteins expression normalized to GAPDH in *tibialis anterior* biopsies obtained from CTR and DM1 patients. Histograms represent mean values and bars represent standard error of the mean (SEM). (**D)** Quantification of proteins expression normalized to GAPDH in *tibialis anterior* and *biceps brachii* biopsies obtained from two DM1 patients who performed two successive biopsies at different years of age. Data obtained from one BB and one TA sample from DM1-1 and DM1-4 were compared with mean values obtained from 2 CTR BB and 3 CTR TA. Bars represent standard error of the mean (SEM). The number of samples analysed in each group (n) is reported in histogram legends. Differences between groups have been evaluated by Student *t*-test. *p<0.05; **p<0.01.

Firstly, the alternative splicing of *INSR* and the expression of several proteins of the insulin pathway was studied in BB biopsies (CTR-1, CTR-2, CTR-3; DM1-1, DM1-2, DM1-3, DM1-4; DM2-1, DM2-2, DM2-3). BB biopsies were performed in DM1 patients for research purposes in order to compare DM1 and DM2 patients. The level of *INSR-B versus INSR-A* expression was determined by RT-PCR analysis (Figs 2A and 3A). *INSR-B* was the predominant isoform expressed in normal skeletal muscle (75.3±1.5%) while *INSR-A* consistently predominated in DM1 skeletal muscle (65.3±14.8%) and an equal amount of *INSR-B* and *INSR-A* expression was observed in DM2 muscle biopsies. In DM patients, even though a low number of samples has been analysed, no relationship seems to be present between the level of *INSR-B* expression and HOMA index, since the insulin resistant patients (DM1-1, DM2-1 and DM2-2) showed the higher levels of *INSR-B* expression (Fig 2A; Table 1).

Western blot analysis has been performed on 4 CTR samples (CTR-1, CTR-2, CTR-3 and CTR-7), 4 DM1 samples (DM1-1, DM1-2, DM1-3 and DM1-4) and 5 DM2 samples (DM2-1, DM2-2, DM2-3, DM2-4 and DM2-5). The results of western blot analysis of insulin pathway (Figs 2B, 2C and 3B) in BB muscles showed that levels of total Akt/PKB, p70S6K and GSK3β expression were not significantly different between DM and controls. On the contrary, ERK1/2 appeared to be higher in DM muscles as compared to controls, however reaching statistical significance only in DM1 (ERK1/2, DM1 *vs* CTR p = 0.02). More interesting, the basal levels of phosphorylation of Akt/PKB (Thr 308) and GSK3β (Tyr 216) were higher in DM muscles compared to CTR, but significantly higher only in DM2 (p-T308-Akt/PKB, DM2 *vs* CTR p = 0.05; p-Y216-GSK3β, DM2 *vs* CTR p = 0.03). Moreover, the basal levels of phosphorylation of p70S6K (Thr 421/Ser 424) and ERK1/2 (Thr 202/Tyr 204) were significantly higher only in DM2 muscles compared to CTR (p-T421/S424-p70S6K, DM2 *vs* CTR p = 0.01; p-T202/Y204-ERK1/2, DM2 *vs* CTR p = 0.05).

Recent studies have indicated that proximal muscles were suitable for biomarker discovery in DM2 but not in DM1 patients [11, 47, 53]. Thus, we performed further analysis to verify if in DM1 patients *INSR* splicing changes and insulin pathway alterations were more evident in TA than in BB. Regarding the *INSR* splicing, no differences in expression levels of the two isoforms were found in CTR subjects between BB (CTR-1, CTR-2) and TA (CTR-4, CTR-5) (CTR BB 71.0±1.4%; CTR TA 69.0±1.4%; Fig 3A). The percentage of *INSR-B* splice form was reduced in all DM1 patients compared to CTR and this reduction was more evident in TA (DM1-1, DM1-4, DM1-5) than in BB (DM1-1, DM1-2, DM1-4) samples (DM1 BB 43.7± 13.1%; DM1 TA 31.7± 3.8%) (Fig 3A). However, a great variability was observed in *INSR* isoform expression levels in DM1 BB muscles given that DM1-2 BB and DM1-3 BB showed *INSR-B* levels similar to those detected in DM1 TA muscles (Figs 2A and 3A). Moreover, no relationship seems to be evident between splicing changes and muscle histopathological alterations since in DM1-1 TA, showing an end-stage abnormal histology, the *INSR-B* expression level was similar to that observed in DM1-4 TA where minimal myopathic changes were present (Figs 1A–1D and 3A).

The western blot analysis of proteins of the insulin pathway showed that in TA the expression of p70S6K was similar in DM1 and in CTR samples, while Akt/PKB and ERK1/2 expression levels were significantly higher in DM1 than CTR (AKT/PKB, CTR vs DM1 p = 0.02; ERK1/2, CTR vs DM1 p = 0.01). Contrary to BB, GSK3β expression was significantly lower in DM1 TA than in CTR TA (p = 0.03). The basal levels of phosphorylation were clearly increased in DM1 TA as compared to CTR TA and this increase was more evident than that observed in BB (Figs 2C and 3C). Indeed, except for p-T421/S424-p70S6K, p-T308-Akt/PKB, p-Y216–GSK3β and p-T202/Y204-ERK1/2 were significantly higher in DM1 TA than in CTR TA (p-T308-Akt/PKB, CTR vs DM1 p = 0.04; p-Y216–GSK3β, CTR vs DM1 p = 0.004; p-T202/Y204-ERK1/2, p = 0.03). When considering the two DM1 patients who performed two successive muscle biopsies, it should be noted that in DM1-1 the alterations of protein expression were similar in BB and in TA or more evident in BB than in TA (Fig 3D). On the contrary, in DM1-4 these alterations were more evident in TA than in BB (Fig 3D). An interesting finding was that differences in protein expression seem to be present between CTR BB (n = 2) and CTR TA (n = 3) (Fig 3D).

Taken together the results of *in vivo* studies suggest that in DM muscle biopsies, increased basal activation of downstream insulin effectors are present compared to CTR, suggesting that further insulin-dependent stimulation of the pathways may be limited. Moreover, these alterations appear to be more evident in distal muscles in DM1 and in proximal muscles in DM2.

### Insulin receptor alternative splicing and insulin signalling activation in muscle cells from DM patients

**Fig 4:**
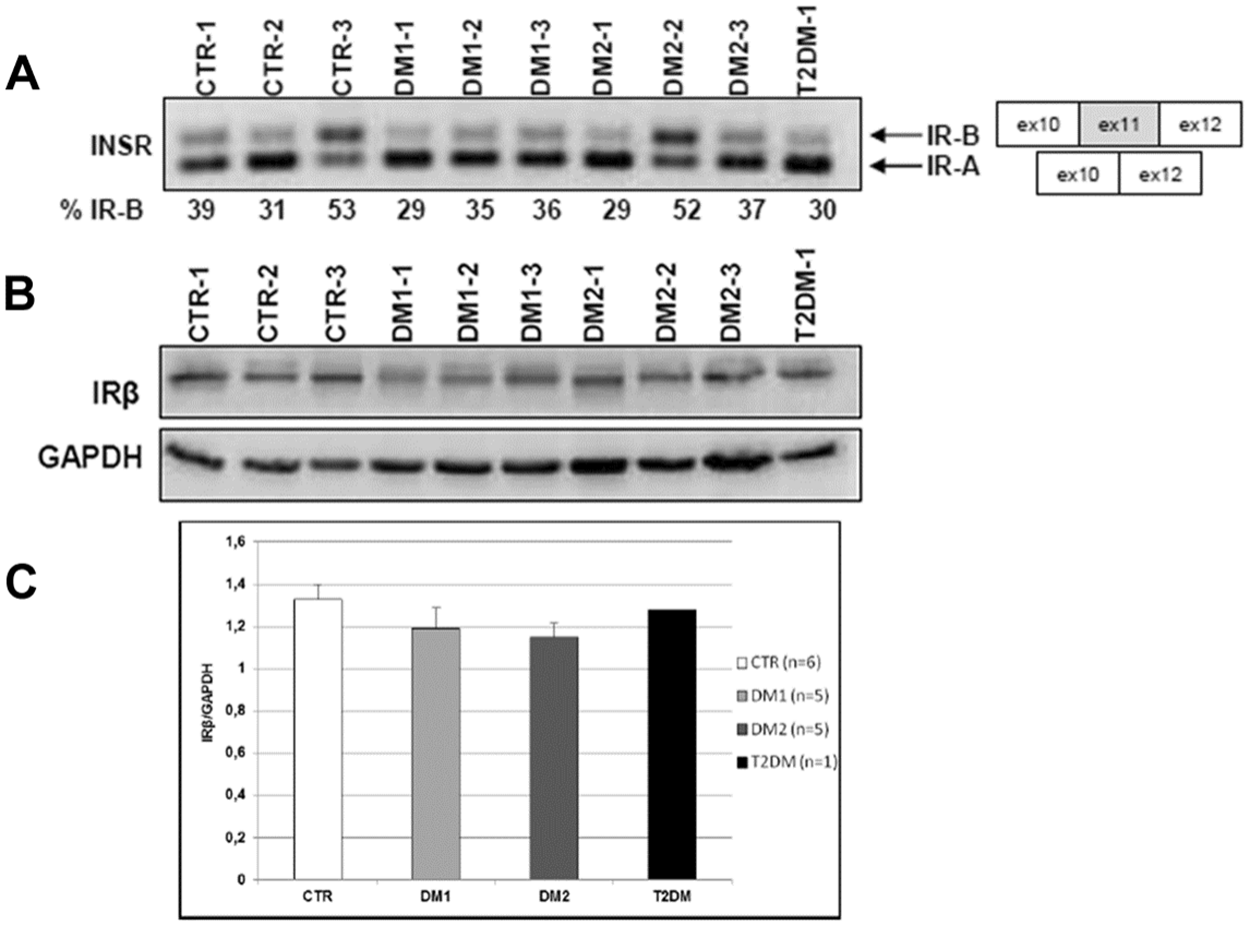
Insulin receptor alternative splicing and protein expression in muscle cells. **(A)** Representative splicing products obtained by RT-PCR amplification of RNA isolated from 5 days differentiated myotubes (T5) obtained from CTR, DM1, DM2 and T2DM patients. Bands were quantified and proportions of isoform *INSR-B* (+exon 11) were calculated. **(B)** Representative western blot analysis of the basal expression of IRβ in myotubes (T5). (**C)** Histograms representing mean values of IRβ expression and bars represent standard error of the mean (SEM). The number of samples analysed in each group (n) is reported in histogram legend. Density of the bands has been normalized to GAPDH expression.

*In vitro* analyses have been performed on muscle cells obtained from 6 CTR (CTR-1, CTR-2, CTR-3, CTR-4, CTR-7 and CTR-8), 5 DM1 (DM1-1, DM1-2, DM1-3, DM1-7 and DM1-8) and 5 DM2 (DM2-1, DM2-2, DM2-3, DM2-4 and DM2-5) patients. RT-PCR analyses have been performed on myotubes from DM1, DM2, CTR and T2DM patients at 5 days (T5) after the addition of differentiation medium to determine the level of *INSR-B versus INSR-A* expression (Fig 4A). As expected, in DM myotubes predominant levels of the *INSR-A* fetal isoform were present (DM1 33.3±3.8%; DM2 39.3±11.7). At this time point of differentiation, a higher expression of *INSR-A* was evident also in CTR and T2DM myotubes (CTR 41.0±11.1; T2DM 30.0±0.0) (Fig 4A). Western blot analysis of IR protein expression by using an antibody against the β subunit of IR showed no differences between DM, T2DM and CTR myotubes (Fig 4B and 4C).

**Fig 5:**
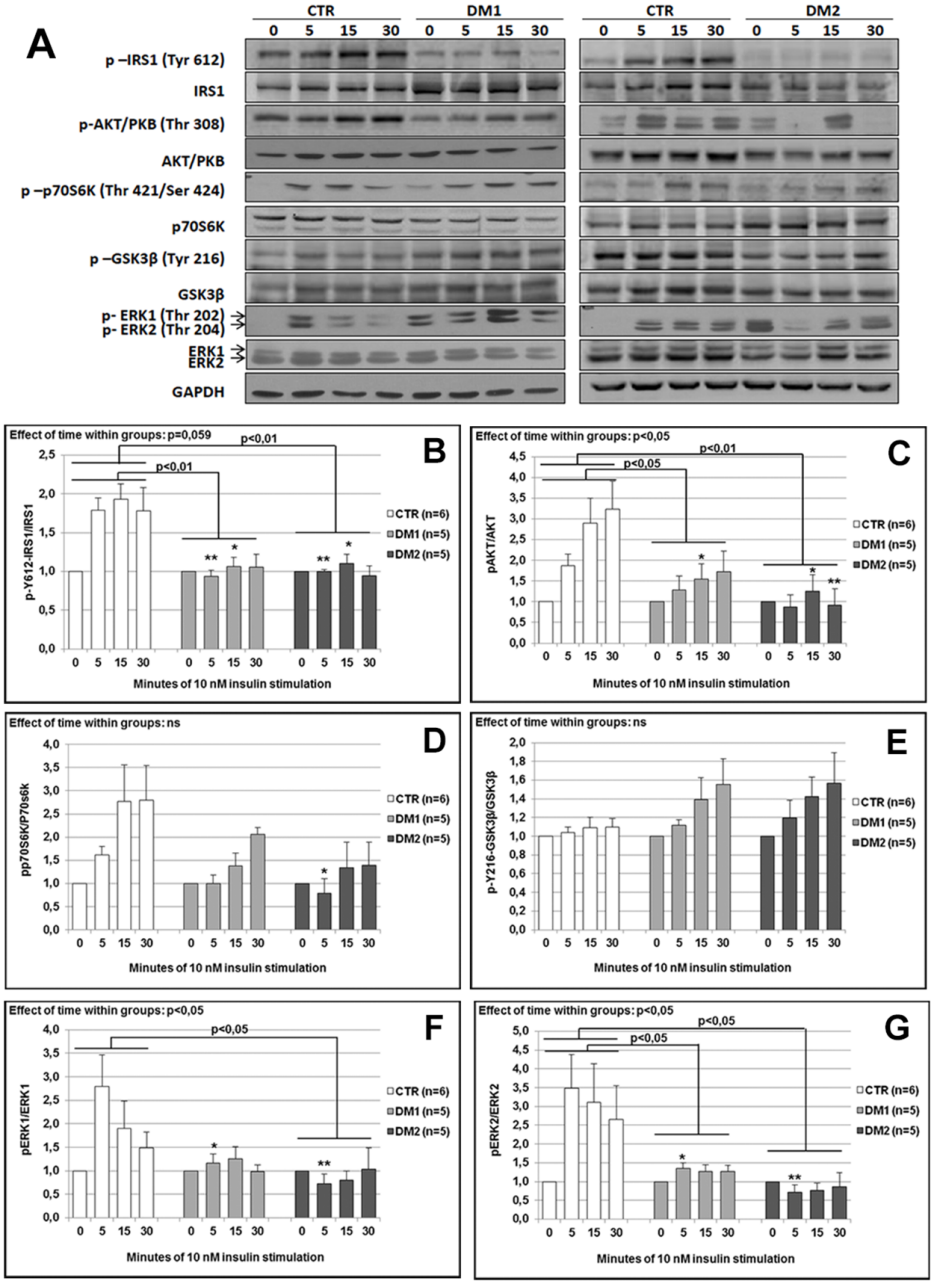
Insulin signalling activation in muscle cells. (**A)** Representative western blot analysis of the expression and activation of proteins involved in the insulin pathway. Myotubes (T5) were cultured in absence or in presence of 10 nM insulin for 5 to 30 minutes. Quantification of IRS1 activation **(B)**, of Akt/PKB **(C)**, p70S6K **(D)**, GSK3β **(E)**, ERK1 **(F)** and ERK2 **(G)**. Histograms represents mean values and bars represent standard error of the mean (SEM). The number of samples analysed in each group (n) is reported in histogram legends. The effect of time within groups was assessed by one-way ANOVA (repeated measures). Results from post-test are marked with connecting capped arcs. *Results from Student *t*-test DM1 or DM2 *versus* CTR at 0-5-15-30 minutes of insulin stimulation: *p<0.05, **p<0.01.

To verify if in DM myotubes post-receptor defects in insulin signalling were present, myotubes at T5 were stimulated with 10 nM insulin at different time points (0, 5, 15 and 30 minutes) based on data previously reported [28]. Expression of total levels of IRS1, Akt/PKB, ERK1/2, p70S6K and GSK3β and of their phosphorylated forms were assessed by western blot analysis (Fig 5). Protein activation was evaluated as phosphorylation/total ratio. To study the activation of IRS1 after insulin stimulation, two different tyrosine phosphorylation sites have been analysed (Y612 and Y896) (Fig 5A and 5B and S1 Fig). Insulin stimulation induced a different Y612-IRS1 activation over time between CTR and DM myotubes (p = 0.059). Indeed, while no insulin activation of IRS1 was evident in DM myotubes, in CTR muscle cells insulin stimulation induced an increase in IRS1 phosphorylation over time (CTR *vs* DM1 p = 0.01; CTR *vs* DM2 p = 0.01). In CTR myotubes, activation of IRS1 was evident starting from 5 minutes and persisted till the end of the experiment (5 minutes: CTR *vs* DM1 p = 0.002, CTR *vs* DM2 p = 0.004; 15 minutes: CTR *vs* DM1 p = 0.01; CTR *vs* DM2 p = 0.02) (Fig 5A and 5B). Similar results have been obtained for Y896-IRS1 activation, however without reaching significance when considering the effect of time within groups (S1 Fig). Nevertheless, a significant lower Y896-IRS1 phosphorylation was observed at 5 and 15 minutes of insulin stimulation in both DM1 and DM2 as compared to controls (5 minutes: CTR *vs* DM1 p = 0.04, CTR *vs* DM2 p = 0.002; 15 minutes: CTR *vs* DM1 p = 0.01, CTR *vs* DM2 p = 0.03). Also for AKT/PKB activation, a significantly different effect of insulin was observed between CTR and DM myotubes over time (p = 0.02) (Fig 5A and 5C). In both CTR and DM1 muscle cells there was a clear trend towards an increased activation of the protein, however this activation was lower in DM1 (CTR *vs* DM1 p = 0.04; 15 minutes: CTR *vs* DM1 p = 0.04). In DM2, no activation of AKT/PKB was observable at all time points considered (CTR *vs* DM2 p = 0.007; 15 minutes: CTR *vs* DM2 p = 0.02; 30 minutes: CTR *vs* DM2 p = 0.01). In both DM and CTR myotubes, insulin induced an increase in p70S6K phosphorylation over time (Fig 5A and 5D). This increase was lower in DM muscle cells compared to CTR, however without reaching significant differences between groups. After insulin stimulation, no increase in GSK3β Y216 phosphorylation was observable in CTR myotubes at all the time points considered (Fig 5A and 5E). On the contrary, in DM1 and DM2 myotubes, a slight increase overtime of GSK3β Y216 phosphorylation was observable starting from 5 minutes. However, no significant differences were reached between groups. In response to insulin, a significant difference in ERK1 and ERK2 activation was observed in DM myotubes as compared to CTR (ERK1, p = 0.02; ERK2, p = 0.02) (Fig 5A, 5F and 5G). Indeed, the increase in ERK1 and ERK2 phosphorylation was evident in CTR myotubes starting from 5 minutes and it persisted till the end of the experiment. On the contrary, on both DM1 and DM2 myotubes no insulin activation of ERK1 and ERK2 was observable during the experiment (ERK1, CTR *vs* DM2 p = 0.03; ERK2, CTR *vs* DM1 p = 0.05, CTR *vs* DM2 p = 0.01).

In comparison, insulin signalling has been studied also in myotubes obtained from 2 T2DM patients. The results showed that, as in DM muscle cells, there is a trend towards a lower or no activation of insulin-associated pathways as compared to CTR (S2 Fig).

When considering the total and phosphorylated expression levels of proteins analysed in basal conditions (no insulin stimulation), only the levels of phosphorylated ERK1/2 were significantly higher in DM and T2DM myotubes as compared to CTR (S3 Fig; p-T202-ERK1: CTR *vs* DM1 p<0.0001, CTR *vs* DM2 p<0.0001, CTR *vs* T2DM p = 0.03; p-Y204-ERK2: CTR *vs* DM1 p<0.0001, CTR *vs* DM2 p<0.0001, CTR *vs* T2DM p = 0.02). Moreover, no differences have been observed in total expression levels between samples except for ERK1/2 (ERK1: CTR *vs* DM1 p = 0.001; ERK2: CTR *vs* DM1 p = 0.001, CTR *vs* DM2 p = 0.01).

**Fig 6:**
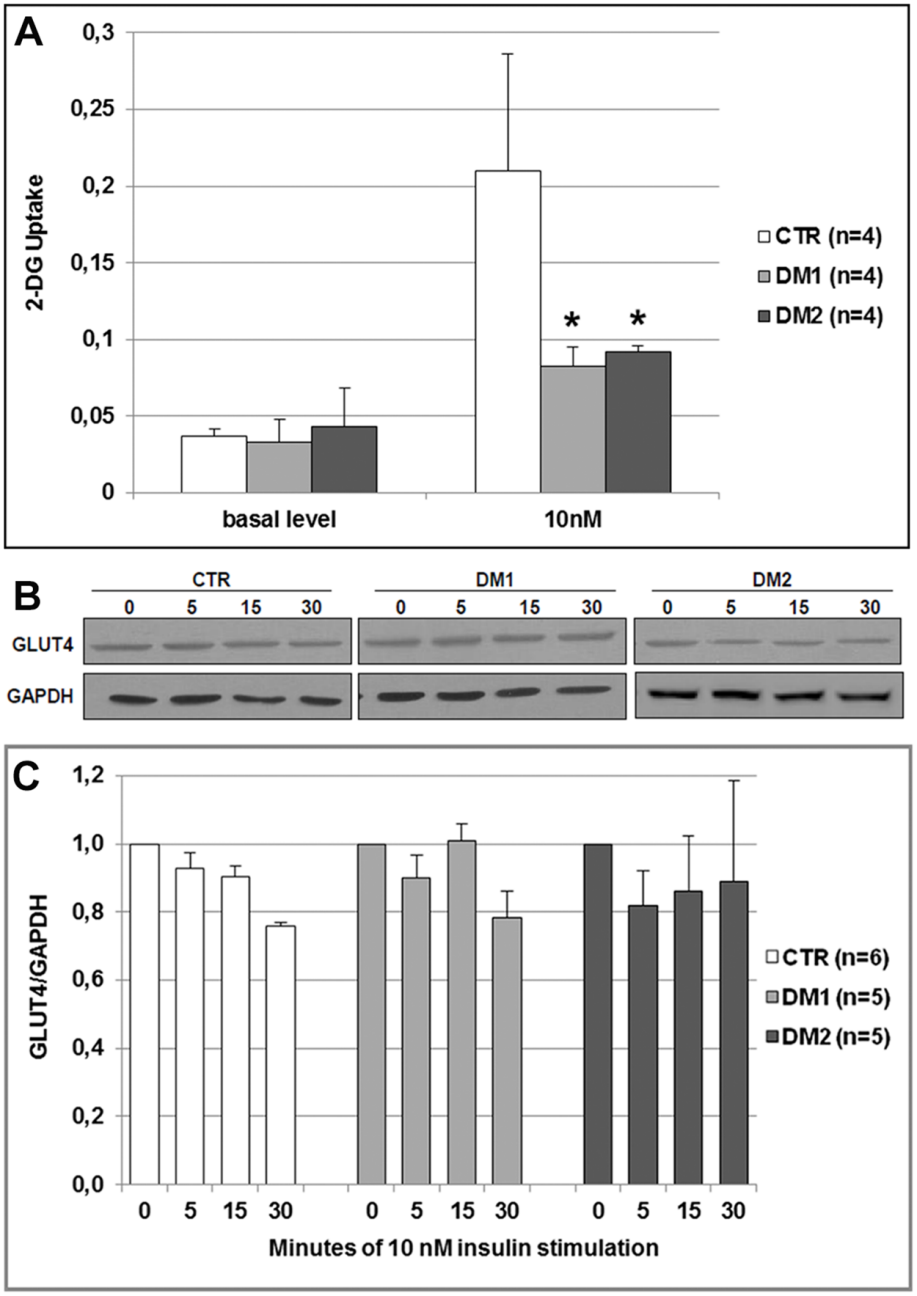
Glucose uptake and GLUT4 expression. (**A)** Histogram representing insulin action on 2-deoxyglucose uptake in human myotubes (T5). Cells were depleted of serum, incubated in absence or presence of 10 nM insulin for 40 min and then 2-deoxyglucose uptake was measured using a colorimetric assay kit. Histograms represent mean values and bars represent standard error of the mean (SEM). (**B)** Representative western blot analysis of the expression of GLUT4 during insulin stimulation. Myotubes (T5) were cultured in absence or in presence of 10 nM insulin for 5 to 30 minutes. Due to the high number of samples analysed, the figure panel is composed by images of bands originating from different gels. Details on how western blot experiments were performed are reported in Materials and Methods. (**C)** Histograms representing mean values of GLUT4 expression analysed by densitometry. Density of the bands has been normalized to GAPDH expression. Bars represent standard error of the mean (SEM). The number of samples analysed in each group (n) is reported in histogram legend. Differences between groups have been evaluated by Student *t*-test. *p<0.05.

Resistance to insulin action in DM myotubes (T5) has been investigated at the level of glucose uptake (Fig 6A). An evident increase of glucose uptake has been observed in CTR myotubes after stimulation with 10 nM insulin, on the contrary, in myotubes from both DM1 and DM2 subjects insulin showed a significant lower stimulatory effect (CTR *vs* DM1 p = 0.04; CTR *vs* DM2 p = 0.05).

Western blot analysis of the insulin dependent glucose transporter GLUT4 did not show any difference in GLUT4 expression during insulin stimulation in both DM and CTR myotubes (Fig 6B and 6C). Moreover, GLUT4 appeared to be similarly expressed in DM and CTR cells.

Taken together the *in vitro* results show that insulin stimulated DM muscle cells exhibit a lower activation of the insulin pathway and a lower glucose uptake compared to CTR myotubes despite the similar *INSR* alternative splicing. These findings suggest that post-receptor defects may contribute to DM insulin resistance.

## Discussion

Decreased insulin sensitivity is a metabolic characteristic of patients with myotonic dystrophies and in the current study we have explored the cellular mechanisms underlying this phenomenon. DM are considered spliceopathies, i.e. several symptoms of DM have been linked to the alteration of alternative splicing in various genes [54]. Insulin resistance has been associated with aberrant splicing of the insulin receptor, however post-receptor defects in insulin signalling have been suggested and cannot be excluded [28–32, 39]. Because a major manifestation of insulin resistance is a decreased insulin-stimulated glucose disposal by skeletal muscle, we have focused our attention on the mechanisms of insulin resistance in this tissue. We have analysed skeletal muscle biopsies and skeletal muscle cell cultures taken from DM1 and DM2 patients and from control subjects with no sign of neuromuscular disease.

In the first part of our study we have analysed *INSR* alternative splicing and insulin pathway in skeletal muscle biopsies from a proximal muscle (BB) and from a distal muscle (TA). As expected, the analysis of *INSR* alternative splicing showed that in BB biopsies the percentage of the *INSR-B* splicing form was reduced in all DM specimens examined [10–11]. However, contrary to data reported by other authors [11, 47], this reduction was more evident in DM1 than in DM2 muscles despite we have analysed a proximal muscle. These data are in line with our previous observations in a different cohort of Italian patients affected by the classical DM1 form and by DM2-PROMM [55] and with those reported on *deltoid* muscle [56]. The difference in IR isoforms expression between DM1 and DM2 BB muscle does not seem to be related to differences in histological alterations since all the DM muscles examined showed the same grade of myopathic changes. Moreover, in a recent work on the comparison in *INSR* splicing changes in type I and II fibers isolated separately from DM1 and DM2 *deltoid* muscles it has been reported that the two fiber types showed an increase of *INSR-A versus INSR-B* expression similar to that observed in the whole muscle. These results suggested that the distinct fiber type involvement in DM1 and DM2 muscle was not related to qualitative differences in *INSR* expression [56]. Western blot analysis of insulin pathway in skeletal muscle showed significant higher basal levels of phosphorylation of Akt/PKB (T308), p70S6K (T421/S424) and ERK1/2 (T202/Y204) in DM2 compared to CTR subjects, while the increase in protein phosphorylation was less evident in DM1 samples. These results are comparable to those reported in skeletal muscle and adipose tissue from insulin resistant and T2DM patients indicating that, as in these patients, also in DM the high levels of basal protein phosphorylation might be accompanied by a lack of further insulin stimulated phosphorylation and thus to a lack of insulin pathway activation [57–59]. These data could explain the impaired glucose utilization observed in DM skeletal muscle [16–19]. It should be noted that the stronger increase in protein phosphorylation in DM2 samples, as compared to DM1 samples, is consistent with the higher risk to develop type 2 diabetes observed in DM2 patients [22]. However, it is possible that in DM1 patients these alterations were less evident since a proximal muscle instead of a distal muscle has been analysed. As previously reported by Jones et al. [32], we have observed an increase in the active form of GSK3β (p-Y216-GSK3β) expression in BB samples from DM1 and DM2 patients. However, this increase was more evident in DM2 where a proximal muscle has been analysed. This result suggest that glycogen synthase and thus glycogen synthesis may also be impaired in DM muscles.

Recent studies have indicated that the distal muscle TA is the best muscle to be used to test therapeutic interventions in DM1 patients because it is preferentially affected at both histological and functional level. Indeed, in DM1 patients splicing events are more severely affected in TA than in proximal muscles such as *vastus lateralis* or *biceps brachii*, indicating that TA biopsies are suitable for biomarker discovery in DM1 [11, 47, 60]. Thus, we have compared *INSR* splicing changes and insulin pathway alterations in TA and in BB of DM1 patients. Consistent with data previously reported [11, 47], the expression of *INSR-A* isoform were slightly higher in TA than in BB samples of DM1 patients. When considering the expression of proteins of the insulin pathway and of their corresponding phosphorylated forms significant differences were evident between DM1 TA and the corresponding CTR. However, the fold changes in protein expression between CTR and DM1 observed in TA were similar to that observed in BB where the differences did not reach statistical significance due to the interindividual variability. An interesting data was that, contrary to DM1 BB, a significantly lower expression of GSK3β was evident in DM1 TA compared to the corresponding CTR. However, a significant difference in GSK3β expression was also evident between CTR BB and CTR TA muscles indicating that this protein could be differently expressed in different muscles. Nevertheless, the active form (p-Y216-GSK3β) expression was increased in both BB and TA samples from DM1 confirming that glycogen synthase and thus glycogen synthesis might be impaired in DM muscles. Taken together, these results seem to indicate that alterations in both *INSR* splicing and insulin signalling are already evident in proximal muscles that are usually less involved in DM1 pathology [2]. An interesting result of this study has been obtained analysing the insulin pathway in proximal and distal muscles of two DM1 patients who performed two successive biopsies at different years of age. In the patient presenting minimal histological changes at BB and TA level, the alterations in *INSR* splicing and protein expression were present in both muscles but more evidently in TA. However, in the other patient where the distal muscle showed severe histopathological changes the alterations of insulin signalling were similar in BB and TA while the *INSR* splicing deregulation was more evident in TA. Even if we have analysed two different muscles at different stage of the disease in only two patients, these data suggest that in DM patients the molecular mechanisms of insulin resistance should not be investigated in severely affected muscles where the loss of muscle fibers is accompanied by an increase of fibrosis. Indeed, it is known that the IGF1/insulin pathway is impaired in dystrophic muscles [61]. Total and phosphorylated levels of proteins of the insulin signalling have been found altered in skeletal muscles of DM1 patients also by Brockhoff et al [40]. However, no information about the muscle analysed and the nutritive status of the patients at the time of biopsy have been reported.

To better understand if the insulin resistance observed in skeletal muscle of our DM1 and DM2 patients may be linked to post-receptor alterations in insulin pathway, we have characterized the expression of key components of the insulin signal transduction pathways during insulin stimulation in myotubes obtained from DM1 and DM2 patients. Human skeletal muscle cells *in vitro* retain a ‘‘memory” of their *in vivo* phenotype when differentiated into myotubes thus representing a useful tool to search for primary defects in skeletal muscle [62]. All analyses have been performed on myotubes at 5 days of differentiation since at this time point both DM and CTR myotubes express the same levels of *INSR* splicing isoforms. Moreover, at this time point of differentiation CTR and DM muscle cells express similar levels of the β subunit of the insulin receptor protein. This allowed us to verify if the insulin insensitivity observed in DM patients is caused by alterations in *INSR* splicing or whether it is caused by post-receptor defects in insulin signalling. The results of our analysis provided evidence for defective insulin signalling in differentiated muscle cells from patients with DM1 and DM2. Indeed, post-receptor signal transduction via both IRS1-Akt/PKB and Ras-ERK pathway appeared to be impaired in DM myotubes which also showed defects in glucose uptake. In DM myotubes a reduction in the magnitude of insulin action or also a delayed onset of insulin action was observable compared to CTR cells. Post-receptor defects were also reported in skeletal muscle of HSA^LR^ mice, where the impairment of AMPK/mTORC1 signalling appears to be independent of insulin receptor deficiency [40].

As for *in vivo* results obtained in BB, the alterations in insulin signalling activation were more evident in DM2 than in DM1 myotubes, confirming at molecular level that this feature of the disease is more severe in DM2 patients [22]. It should be noted that, considering the basal level of protein phosphorylation, the insulin pathway seemed to be already active in DM myotubes compared to CTR, confirming our data obtained *in vivo*. However, the basal levels of glucose uptake were similar in DM and CTR cells, indicating that even if the DM pathway seems to be more active at basal level, no overall increase in glucose uptake was observed. Thus, the results obtained from *in vitro* and *in vivo* studies, strengthen the hypothesis that the higher basal activation of the insulin signalings may be accompanied by a lack of further stimulation of the pathways and hence may contribute to insulin resistance [57–59]. As to concern GSK3β, the increase in the expression of the active p-Y216-GSK3β in DM myotubes support our data on DM muscle biopsies. Our results obtained in DM1 and DM2 muscle cells were supported by data obtained in T2DM myotubes which also do not show signalling activation after insulin stimulation as reported by other authors [63].

In line with data previously reported by other authors for DM1 [10, 28], our results on glucose uptake demonstrated that stimulation with 10 nM of insulin induced a lower increase of glucose uptake in both DM1 and DM2 myotubes compared to CTR. We thus examined whether GLUT4 expression was altered in DM myotubes. Our results did not indicate differences in GLUT4 expression between DM and CTR skeletal muscle cells, thus indicating that impaired glucose uptake attributed to insulin resistance cannot be explained by a defective synthesis of the transporter. Insulin stimulation induces redistribution of GLUT4 from a perinuclear storage vesicular compartment to the cell surface, where glucose uptake takes place. Thus, defects in insulin stimulated translocation of GLUT4 storage vesicles as well as perturbation of microtubule reorganization and of actin remodelling could be hypothesized [64–66].

In conclusion, although a limit of this study is that we have compared TA and BB muscles from different patients or from same patients but at different stages of disease, our *in vivo* results seem confirm that distal muscles in DM1 and proximal muscles in DM2 are suitable for biomarker discovery [11, 47, 60]. However, in DM1 patients alterations in *INSR* splicing insulin signalling are already evident in BB suggesting that proximal muscle should be used for biomolecular studies when distal muscle are severely affected. Moreover, our results indicate that post-receptor defects in insulin signalling may contribute to insulin resistance in DM patients despite the aberrant alternative splicing of *INSR* gene. Further analysis will be necessary to identify the precise molecular defects in insulin signalling that cause muscle insulin resistance in these two dystrophic myotonic disorders. It is likely that metabolic changes contribute to muscle weakness and wasting, cardiovascular diseases and neuropathies, thus, the identification of therapeutic target for insulin resistance treatment in DM patients could contribute to ameliorate the multisystemic spectrum of these diseases.

## Supporting information

S1 Table Clinical data on CTR and DM patients used in this study. (DOC)

S1 Fig Y896 IRS1 activation in DM skeletal muscle cells. (**A)** Representative western blot analysis of Y896-IRS1 activation in DM1 and DM2 myotubes compared to controls. Myotubes (T5) were cultured in absence or presence of 10 nM insulin for 0 to 30 minutes. (**B)** Quantification of IRS1 activation. Histograms represents mean values analysed in 6 CTR and 2 T2DM. Bars represent standard error of the mean (SEM). The number of samples analysed in each group (n) is reported in histogram legends. The effect of time within groups was assessed by one-way ANOVA (repeated measures). *Results from Student *t*-test DM1 or DM2 *versus* CTR at 0-5-15-30 minutes of insulin stimulation: *p<0.05, **p<0.01.
(TIF)

S2 Fig Insulin signalling activation in T2DM myotubes. (**A)** Representative western blot analysis of the expression and activation of proteins involved in the insulin pathway in T2DM myotubes compared to controls. Myotubes (T5) were cultured in absence or presence of 10 nM insulin for 0 to 30 minutes. Quantification of IRS1 **(B-C)**, of Akt/PKB **(D)**, p70S6K **(E)**, ERK1 **(F)**, ERK2 **(G)** and GSK3β **(H)** activation. Bars represent standard error of the mean (SEM). The number of samples analysed in each group (n) is reported in histogram legends.
(TIF)

S3 Fig Basal expression levels in skeletal muscle cells. Quantification of basal protein expression normalized to GAPDH. Bars represent standard error of the mean (SEM). The number of samples analysed in each group (n) is reported in histogram legends. Differences between groups have been evaluated by Student *t*-test. *p<0.05, **p<0.01, ***p<0.001, ****p<0.0001.
(TIF)
