# Supplementary material for: Receptor and post-receptor abnormalities contribute to insulin resistance in myotonic dystrophy type 1 and type 2 skeletal muscle
Source: PLoS One. 2017 Sep 15;12(9):e0184987. doi: 10.1371/journal.pone.0184987 (PMC5600405; doi:10.1371/journal.pone.0184987)
Supplement: S1 Table — (DOC) [file pone.0184987.s001.doc]

| **Patient** | **Elbow flexors MRC-R (I/II)^a^** | **Elbow flexors MRC-L (I/II)** | **Hip flexors MRC-R (I/II)** | **Hip flexors MRC-L (I/II)** | **Feet dorsi- flexors MRC-R (I/II)** | **Feet dorsi- flexors MRC-L (I/II)** | **Other comorbidities** | **Pharmacologic treatment** |
| --- | --- | --- | --- | --- | --- | --- | --- | --- |
| **CTR-1** | 5 | 5 | 5 | 5 | 5 | 5 | none | none |
| **CTR-2** | 5 | 5 | 5 | 5 | 5 | 5 | none | none |
| **CTR-3** | 5 | 5 | 5 | 5 | 5 | 5 | none | none |
| **CTR-4** | 5 | 5 | 5 | 5 | 5 | 5 | none | none |
| **CTR-5** | 5 | 5 | 5 | 5 | 5 | 5 | none | none |
| **CTR-6** | 5 | 5 | 5 | 5 | 5 | 5 | none | none |
| **CTR-7** | 5 | 5 | 5 | 5 | 5 | 5 | none | none |
| **CTR-8** | 5 | 5 | 5 | 5 | 5 | 5 | none | none |
| **DM1-1** | 4/4.7 | 4/4.7 | 4/4 | 4/4 | 4.3/3 | 4.3/3 | Hypothyroidism well controlled by pharmacologic therapy; hypertension | Valsartan; levotiroxin; amlodipine |
| **DM1-2** | 4 | 4 | 5 | 5 | 4.7 | 4.7 | none | none |
| **DM1-3** | 5 | 5 | 5 | 5 | 4.3 | 4 | none | none |
| **DM1-4** | 5/5 | 5/5 | 5/5 | 5/5 | 5/5 | 5/5 | none | Mexiletine |
| **DM1-5** | 5 | 5 | 5 | 5 | 4.3 | 4.3 | none | Mexiletine |
| **DM1-6** | 4.7 | 4.7 | 5 | 5 | 5 | 5 | none | none |
| **DM1-7** | 4.3 | 5 | 5 | 5 | 4 | 3.6 | Hypothyroidism well controlled by pharmacologic therapy | Levotiroxin |
| **DM1-8** | 5 | 5 | 5 | 5 | 3 | 3.6 | Dyslipidemia | Colestyramine |
| **DM2-1** | 4 | 4 | 3 | 3 | 1 | 2.7 | Hypothyroidism well controlled by pharmacologic therapy; hypertension; type 2 diabetes | Ramipril; levotiroxin; sotalol; ticlopidine; fenofibate; metformin; acarbose |
| **DM2-2** | 4.3 | 5 | 4.3 | 4.3 | 5 | 5 | Hypertension | Zofenopril |
| **DM2-3** | 5 | 5 | 5 | 5 | 5 | 5 | none | none |
| **DM2-4** | 5 | 5 | 4 | 3.6 | 5 | 5 | Hypertension | Ramipril |
| **DM2-5** | 5 | 5 | 4.3 | 4.3 | 5 | 5 | none | none |

**Table 1: Clinical data on CTR and DM patients used in this study.**

CTR, control; DM1, myotonic dystrophy type 1; DM2, myotonic dystrophy type 2; F, female; M, male.

^a^Clinical data reported are relative to the time at the first biopsy (I) and at the second biopsy (II).
