# Supplementary figures and images for: Receptor and post-receptor abnormalities contribute to insulin resistance in myotonic dystrophy type 1 and type 2 skeletal muscle
Source: PLoS One. 2017 Sep 15;12(9):e0184987. doi: 10.1371/journal.pone.0184987 (PMC5600405; doi:10.1371/journal.pone.0184987)

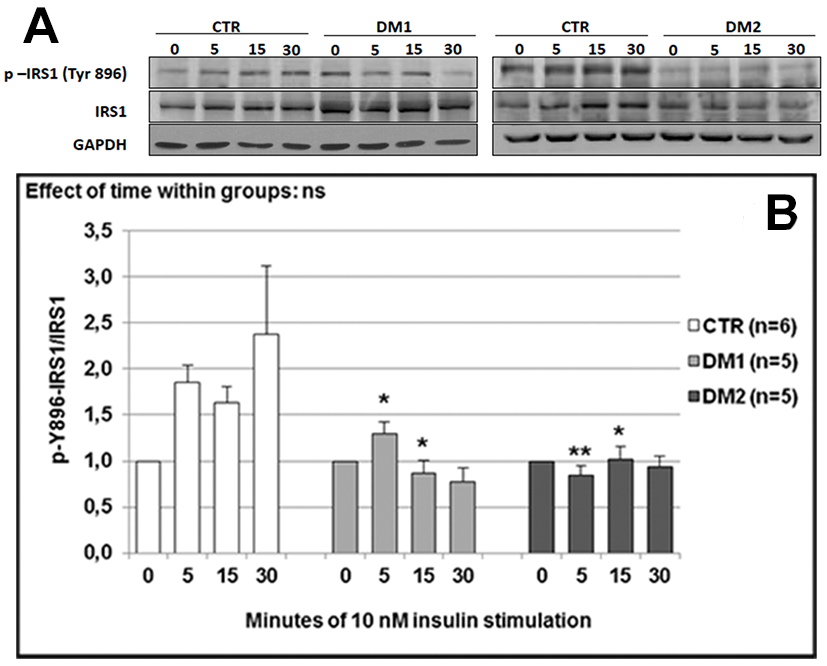

Supplement: S1 Fig — (A) Representative western blot analysis of Y896-IRS1 activation in DM1 and DM2 myotubes compared to controls. Myotubes (T5) were cultured in absence or presence of 10 nM insulin for 0 to 30 minutes. (B) Quantification of IRS1 activation. Histograms represents mean values analysed in 6 CTR and 2 T2DM. Bars represent standard error of the mean (SEM). The number of samples analysed in each group (n) is reported in histogram legends. The effect of time within groups was assessed by one-way ANOVA (repeated measures). *Results from Student t-test DM1 or DM2 versus CTR at 0-5-15-30 minutes of insulin stimulation: *p<0.05, **p<0.01. (TIF) [file pone.0184987.s002.tif]

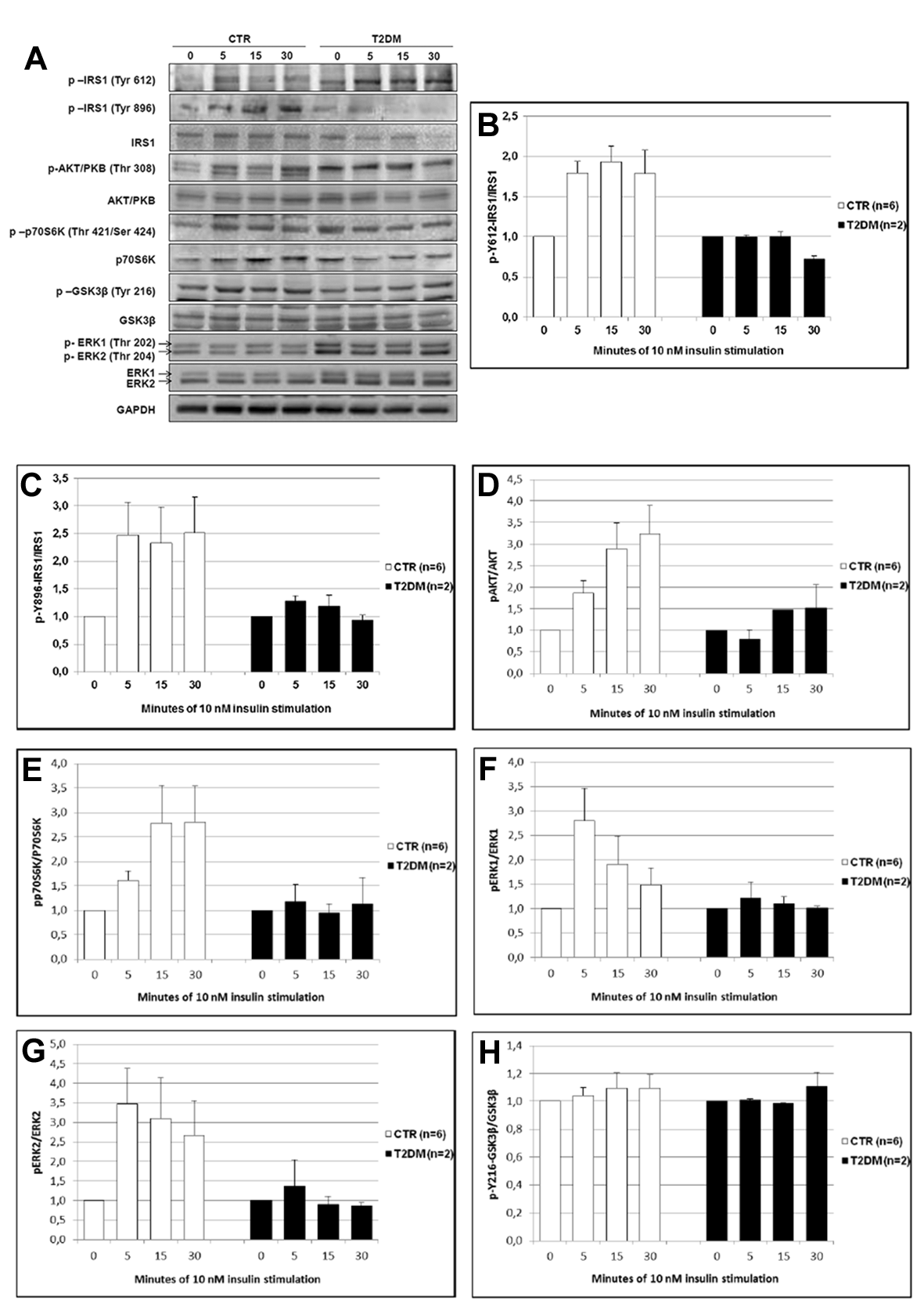

Supplement: S2 Fig — (A) Representative western blot analysis of the expression and activation of proteins involved in the insulin pathway in T2DM myotubes compared to controls. Myotubes (T5) were cultured in absence or presence of 10 nM insulin for 0 to 30 minutes. Quantification of IRS1 (B-C), of Akt/PKB (D), p70S6K (E), ERK1 (F), ERK2 (G) and GSK3β (H) activation. Bars represent standard error of the mean (SEM). The number of samples analysed in each group (n) is reported in histogram legends. (TIF) [file pone.0184987.s003.tif]

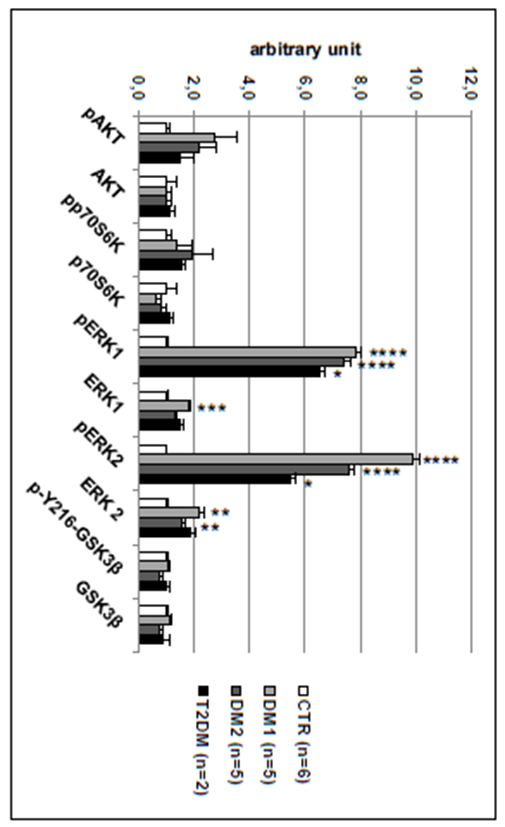

Supplement: S3 Fig — Quantification of basal protein expression normalized to GAPDH. Bars represent standard error of the mean (SEM). The number of samples analysed in each group (n) is reported in histogram legends. Differences between groups have been evaluated by Student t-test. *p<0.05, **p<0.01, ***p<0.001, ****p<0.0001. (TIF) [file pone.0184987.s004.tif]
